# A Review of Applications of Artificial Intelligence in Gastroenterology

**DOI:** 10.7759/cureus.19235

**Published:** 2021-11-03

**Authors:** Khalid Nawab, Ravi Athwani, Awais Naeem, Muhammad Hamayun, Momna Wazir

**Affiliations:** 1 Internal Medicine, Penn State Holy Spirit Hospital, Camp Hill, USA; 2 Internal Medicine, Khyber Medical University, Peshawar, PAK; 3 Management Sciences, Bacha Khan University, Charsadda, PAK; 4 Internal Medicine, Hayatabad Medical Complex, Peshawar, PAK

**Keywords:** artificial neural networks, artificial intelligence in gastroenterology, artificial intelligence in medicine, deep learning artificial intelligence, clinica gastroenterology

## Abstract

Artificial intelligence (AI) is the science that deals with creating ‘intelligent machines’. AI has revolutionized medicine because of its application in several fields across medicine like radiology, neurology, ophthalmology, orthopedics and gastroenterology. In this review, we intend to summarize the basics of AI, the application of AI in various gastrointestinal pathologies till date as well as challenges/ problems related to the application of AI in medicine. Literature search using keywords like artificial intelligence, gastroenterology, applications, etc. were used. The literature search was done using Google Scholar, PubMed and ScienceDirect. All the relevant articles were gathered and relevant data were extracted from them. We concluded AI has achieved major feats in the past few decades. It has helped clinicians in diagnosing complex diseases, managing treatments as well as in predicting outcomes, all in all, which helps doctors from all over the globe in dispensing better healthcare services.

## Introduction and background

The term artificial intelligence (AI) was coined by a computer scientist, John McCarthy in 1956. It is the science that deals with creating machines with intelligence comparable to that of a human. It is concerned with developing such modernized computer programs that have the capability to resemble and imitate an actual sapiens’ intelligence. That’s the reason why these machines are also sometimes referred to as ‘intelligent machines’.

AI, although being one of the most talked-about topics in recent years, can be referenced back to the 1950s. A British mathematician, Alan Turing with the help of his very famous ‘Turing Test’, displayed impeccable human-like intelligent behavior by a computer [1]. Ever since then, it has been used in several fields. The last few decades have shown a rapid surge in interest when it comes to its use in medicine. Various AI-based algorithms have been approved by the Food and Drug Administration.

AI has without any doubt revolutionized medicine. The first-ever application of AI in Surgery was recorded in 1976 wherein acute abdominal pain was diagnosed with the help of computer analysis [2]. Nonetheless, its application in fields like radiology, orthopedics, neurology, pathology, ophthalmology and gastroenterology has been absolutely remarkable.

In this review, we intend to highlight the basics of AI, with a focus on applications of AI in gastrointestinal medicine. We also examine the challenges involved in the applications of AI in GI medicine.

## Review

Methodology

This review was done using current and older literature that was available on AI and its role in gastroenterology. We did not restrict our time frame of literature publication; however, at the time of literature search, articles published till the end of January 2021 were reviewed. The following databases have been searched for relevant articles: Google Scholar, PubMed, and ScienceDirect.

The researches were identified using the keywords: Artificial Intelligence, Gastroenterology, and Application of AI. Additional articles from the primary article were also explored and cited wherever relevant.

Inclusion criteria included articles focusing on the application of AI only in gastroenterology. Articles with keywords mentioned above were reviewed. Only the articles falling under the inclusion criteria were selected. The articles explaining the role of AI in other body systems were excluded.

All the articles explaining the basics of Artificial Intelligence were thoroughly investigated and chosen. Application of AI in various gastrointestinal pathologies was researched and relevant information was extracted and added by reading full articles.

Basics of AI

AI includes a wide variety of methodologies and algorithms. In supervised learning, the algorithms are referred to as ‘supervised’ because they are literally supervised by an external agent, in terms of providing it with the expected output. Therefore both the input data and the expected output are provided and the machine then learns from that data how to reach the expected output [3]. Examples of Supervised learning include decision trees, support vector machines (SVM), artificial neural networks (ANNs), etc. [4].

In unsupervised learning, the learning is not supervised as the expected output is not provided and the algorithm works only on the basis of the input data by identifying trends in it [3]. Examples of Unsupervised learning include k-means and Kernel density estimation [5].

In reinforcement learning, the algorithms interact with the environment so there exists a feedback loop between the learning system and its experiences [3]. It is similar to a supervised learning system in a way that there’s an external interaction; however, this interaction turns out to be noisy and not as effective as it is seen in supervised learning. Examples: Q-learning, temporal-difference learning, deep reinforcement learning, etc.

Currently, the most common AI approaches used in medicine are artificial neural networks (ANNs), support vector machines (SVM), fuzzy expert systems, the Bayesian approach, and hybrid intelligent systems.

An ANN is a bunch of interconnected processing units that simulate the existence of the complex neural network existing in the human brain. The very idea of forming such a multilayered complex network was inspired by Frank Rosenblatt who introduced the concept of perceptron [6]. A perceptron is a unit that imitates the actual neuron in our brain. The first artificial neuronal network was created by McCulloch and Pitts in 1943 using simple binary threshold functions [7]. The ANN basically consists of an input layer, output layer and one or more hidden layers that exist between both the layers. A deep neural network is a form of ANN with multiple hidden layers in the network. It has a deeper architecture as compared to the traditional ANNs and machine learning through the use of deep neural networks is referred to as deep learning (DL). A convolutional neural network (CNN) is a type of ANN that is most popular for image processing [8].

The SVM is the most popular classification algorithm, invented by Vladimir N Vapnik and Alexey Ya Chervonenkis in 1963, and was widely accepted and used before the development of DL [9]. The fuzzy logic has been explored widely in medicine. Unlike conventional systems like Boolean, the fuzzy logic allows the existence of a wide range of responses, from 0 to 1. This system was proposed by Lofti Zadeh in 1965 [10]. The Bayesian model is a typical classification algorithm that works on the basis of probability distribution [11].

Role of AI in gastrointestinal pathology

The advent of Artificial Intelligence has been proven to be very helpful for clinicians to make diagnosis, provide treatment as well as predict outcomes especially when it comes to GI pathologies. Here are some of the most common applications of AI in gastroenterology:

Barrett’s Esophagus

In a recent study, an algorithm was developed based on the specific texture, color filters and ML with a sensitivity and specificity of 83% [12]. This algorithm was able to diagnose early neoplastic lesions from endoscopic images with a decent accuracy further implying its use in clinical practice.

Another study was done using the encoder-decoder artificial neural network wherein the sensitivity, specificity, and accuracy achieved was 83.7%, 100%, and 89.9% respectively [13]. This study was a follow-up on their prior publications which were also about the application of AI and DL in the evaluation of Barett’s esophagus [14,15].

Esophageal Cancer

Esophageal cancer is a highly aggressive cancer with a very poor prognosis in its advanced stages. Esophagogastroduodenoscopy is the gold standard method for detecting esophageal cancer but these lesions can be easily overlooked by endoscopists, especially in white-light imaging [16]. Due to advancements in AI, there are several studies that encourage the use of AI systems in diagnosing esophageal cancer including both, squamous cell carcinoma and adenocarcinoma [15-17].

In an article published by Yoshimasa Horie in Japan, 1118 test images were analysed in 27 seconds through CNN and were correctly diagnosed with esophageal cancers with a sensitivity of 98% [18]. It could even detect cancer lesions less than 10 mm in size. Superficial esophageal cancer was distinguished from advanced cancer with an accuracy of 98%.

A system of computer-aided detection (CAD) using a deep neural network (DNN) was developed by Cai et al in 2019 [19]. In this study, early esophageal squamous cell carcinoma was diagnosed using the DNN-CAD system and by the conventional method. The sensitivity and accuracy turned out to be higher in the DNN-CAD system than in conventional endoscopy.

Recently a single-shot multibox detector was developed using a CNN in order to diagnose various histological grades of esophageal neoplasms [20]. In this study, 936 endoscopic images (498: white-light imaging images; WLI and 438 narrow-band imaging images; NBI) were used. This AI system analysed 264 images in just 10 seconds with the sensitivity and specificity of 96.2% and 70.4%, respectively. The accuracy of this system in differentiating the esophageal neoplasms as per their histological grades turned out to be 92%. Also, a better diagnostic accuracy in NBI (95%) than in WLI (89%) images was seen.

Gastric Cancer

The diagnosis of gastric cancer again uses an integrated approach but is synonymous with the usual endoscopy. Integration of DL into the diagnostic process of gastric cancer has now become the gold standard for detecting gastric cancer [21].

AI in Endoscopy:

Hirasawa et al reported the application of CNN-based system to detect gastric cancer in endoscopic images, in January 2018 [22]. This diagnostic system, based on Single shot MultiBox Detection architecture, was trained using 13,584 high-resolution endoscopic images of gastric cancer. 2296 test images were analysed in 47 seconds out of which, CNN successfully diagnosed 71/77 cancer lesions. The overall sensitivity in this study was 92.2% with a predictive value of 30.6% since 161 non-cancerous lesions were detected as gastric cancers. This caused misdiagnosis of gastric cancer.

In a study by Ueyama et al. in 2021, AI-assisted CNN CAD system was developed on the basis of magnifying endoscopy with narrow-band imaging (ME-NBI) images so as to diagnose gastric cancer [23]. A dataset of 5574 ME-NBI images was used. 2300 test images were analysed in 60 seconds with the sensitivity, specificity and accuracy of 98%, 100%, and 98.7%, respectively.

Gastrointestinal Artificial Intelligence Diagnostic System (GRAIDS) was a method developed for the diagnosis of upper gastrointestinal cancers in a study proposed by Luo et al in December 2019 [24]. The sample size in this study involved only those patients that were histologically proven to suffer from malignant lesions. The diagnostic performance of GRAIDS was assessed with the help of an internal and prospective validation set from Sun Yat-sen University Cancer Centre and additional external validation sets from five different primary care hospitals in China. The diagnostic accuracy turned out to be 95%: internal validation set, 92.7%: prospective set and lied between 91.5%-97.7%: five external validation sets. The diagnostic accuracy was comparatively higher in GRAIDS, the sensitivity being similar to that of expert endoscopists and higher as compared to non-expert endoscopists.

A deep convolutional neural network (DCNN) was developed by Wu et al., in 2019 to detect early gastric cancer without blind spots, with an accuracy, sensitivity and specificity of 92.5%, 94% and 91% [25].

Another AI-based CNN-CAD system was devised to determine the invasion depth of gastric cancer [26]. It is crucial to accurately predict the invasion depth so as to effectively screen patients for endoscopic resection. Endoscopic resection is done for patients in whom the early gastric invasion depth is within the mucosa or submucosa of the stomach wall. The sensitivity, specificity, and accuracy was 76.47%, 95.56%, and 89.16%, respectively [26].

A CNN model was also developed in diagnosing cirrhotic patients based on ultrasound images [27]. A support vector machine classifier is then used to classify the samples into normal or abnormal cases. The results obtained through this study have shown to help extract the liver capsules and make accurate diagnosis [27].

AI in MRI/CT Imaging:

Application of AI in MRI/ CT in diagnosing gastric cancers isn’t as extensive as that of endoscopy however they can sometimes be helpful in detecting gastric cancer. In a study by Yu et al. CT plays a role in predicting T stage in patients suffering from gastric cancer [28].

AI in Pathological Diagnosis:

A pathological approach will definitely aid the clinicians in diagnosing as well as planning the treatment for the patient however this area hasn’t been delved into yet. Future concrete studies are necessary to substantiate the role of AI in relation to pathological diagnosis. Currently, morphologic diagnosis is still the gold standard in diagnostic pathology, however, its main limitation is diagnostic variability in bearing error among pathologists. Therefore, algorithmic intelligence was introduced into the pathology domain, and more specifically into the morphological analysis of cells and tissues. With the help of digital pathology equipment including microscopic cameras and whole slide imaging scanners, morphology-based automated pathologic diagnosis has become a reality. Typical digital image analysis tasks in diagnostic pathology include segmentation, detection, and classification, in addition to quantification and grading [29]. For example, in the diagnosis of gastric cancers, AI has proved a potential benefit with enhanced image segmentation and reduced diagnostic time. Recently, Li et al. suggested the new GastricNet, a DL-based framework, which works with an accuracy of 100% on gastric pathological slices [30].

Helicobacter pylori Infection

H. pylori infection is strongly associated with peptic ulcers and gastric cancers [31,32]. AI coupled with endoscopy has been used to aid in diagnosis of H. Pylori infection.

According to a study proposed by Shichijo et al. in 2017, a first, 22-layer deep CNN was constructed, trained, and fine-tuned with the help of a dataset of 32,208 patients (including images that are positive and negative for H. Pylori) [33]. Second CNN was trained using images that were classified as per the anatomical locations. 11,481 images from 397 patients were then analyzed and evaluated by the CNN as well as the endoscopists, to check its potential in diagnosing H. Pylori infection, the results of both of which were compared later. The sensitivity, specificity and accuracy for the first CNN were 81.9%, 83.4% and 83.1%, respectively. The sensitivity, specificity and accuracy for the second CNN were 88.9%, 87.4% and 87.7% respectively. The same values for skilled endoscopists were 79%, 83.2% and 83.4%, respectively. As per the results, secondary CNN can be used as a valuable tool to diagnose H. Pylori infection since it has a significantly higher accuracy as compared to endoscopists [33].

A similar paper was published by Itoh et al. in 2018 wherein a CNN was developed to make early diagnosis of H. Pylori infection so that gastric cancer can be prevented [34]. The sensitivity and specificity for this model were 86.7% and 86.7%, respectively, with area under the curve being 0.956 [34].

An algorithm was designed using blue laser imaging (BLI)-imaging and linked color imaging (LCI) by Nakashima et al. in 2018 [35]. Sample size in this study was 222 (105 H. Pylori positive patients). 3 still images were taken by the endoscopist with the help of white light imaging (WLI), BLI-bright, and LCI. The area under the curve for each of them was 0.66, 0.96 and 0.95, respectively [35].

Polyps and Colon Cancer

Polyps or colonic polyps are extra growths that occur on the surface of the inner lining of the colon. It is usually believed that a huge percentage of people suffering from colonic polyps, especially adenomatous polyps, end up developing colorectal carcinoma (CRC). Colonoscopy is considered as the gold standard for reducing the mortality due to CRC [36,37].

In a study proposed by Misawa et al. [34] that an AI-based system can potentially provide a system of automatic detection of polyps and such systems will likely fill the gap between endoscopists with various levels of experience [38].

Deep CNNs were designed and trained using 8,641 hand-labeled images and 20 colonoscopy videos by Urban et al. [39] The accuracy turned out to be 0.991 and the AUROC was 96.4%.

Keeping in mind that Narrow-Band Imaging (NBI) has the potential to differentiate between neoplastic and non-neoplastic colorectal polyps, a computer-based method for classifying colorectal polyps was devised in 2011 [40]. Sample size involved 214 patients with colorectal polyps. After assessing them with zoom NBI colonoscopy, the computer-based algorithm revealed a specificity, sensitivity, and accuracy of 90.3%, 90.5%, and 93.1%, respectively, which was comparable to that of the expert group. The results were definitely higher than that of the non-expert group [40].

Ultra-high magnification endocytoscopy is a CAD system that has been shown to be helpful in differentiating invasive colorectal cancer from non-invasive lesions in a paper published by Takeda et al. [41] 5543 endocytoscopy from 238 lesions were used for training the algorithm. The sensitivity, specificity, and accuracy were 89.4%, 98.9%, and 94.1%, respectively [41].

Discussion

From the review of the literature, we concluded the following trends (Table 1):

**Table 1:** Conclusions from literature review.

Major conclusions from literature review
Most modalities that have achieved high accuracy are based on computer vision analyzing imaging data.
The imaging data can be endoscopic images, ultrasound images, radiological images or histopathological images.
AI can identify lesions that may potentially be missed by the human eye as AI will analyze the image pixel by pixel whereas a human may miss spots if attention is not paid.
AI can accelerate or assist in not only the identification of pre-cancerous/cancerous lesion, but also identify H. pylori infection, identify a cirrhotic liver and classify polyps.

AI is transforming various industries and healthcare is no exception. It is emerging as an effective tool that is assisting healthcare providers in making their job a lot more efficient. It is especially finding a place in GI because of the number of screening and diagnostic procedures performed in GI. It seems like image processing and computer vision (CV) technologies are the specifically popular aspects of AI that are becoming more and more useful to GI professionals. It makes sense though. GI professionals rely heavily on imaging modalities like CT scans and MRI as well as visual data from procedures like endoscopies. Histopathology also plays a vital role in diagnostics. Most of these modalities rely on the expertise of the provider interpreting these images. The human eye may miss portions of the image that contains the specific lesion. This, however, is not a problem with AI, since each pixel of an image in each frame of a video is analyzed and a well-trained algorithm will not miss any of such lesions.

However, AI relies heavily on the data provided. Any AI model is as good as the quality and amount of data used to train that model. Therefore, it is imperative to collect, clean and label good quality and quantity of data to train any model. Furthermore, a model trained on data collected through a specific device may not work that well on input from a different device as noise filters or color filters may cause changes in the value of pixels leading to an input that is very different from the data used to train the model. 

Even if we have a well-trained model, considering the ethical and legal liabilities involved in case a lesion is missed, relying on AI solely will not be wise. Also, the imaging data from endoscopies still requires a skilled clinician to perform the procedure to capture the data. Therefore, at least in the near future, AI will likely be used as an assistive tool rather than replace the human eye. Further, although AI can potentially make better diagnoses than humans, it cannot possibly replace the personal aspects that any doctor-patient relationship has such as compassion, empathy and effective communication. Therefore contrary to popular media opinion that AI will replace a lot of doctors, it does not seem like we are close to that yet.

One specific procedure where AI can have more importance than others is likely wireless capsule endoscopy which involves the swallowing of a capsule equipped with a camera. The camera captures images as it passes through the GIT. These images are then downloaded and studied by a clinician. This is a procedure that does not rely on the skill of the clinician. It also captures large amount of data for a single patient going through which can be exhaustive for the clinicians therefore, by automating the interpretation of the images from a wireless capsule endoscopy, we can make the process a lot more efficient. An efficient hypothetical model would be AI flagging studies that are likely abnormal and prioritize them for the human interpreter over studies that the AI believes is normal. 

As is obvious from the review of literature, most of the work is done on screening and diagnostic modalities in GI. This is because of the excellent feats of computer vision achieved by CNN. However, there are many other aspects where there is potential. This could be in the form of diagnosis or risk assessment from textual data like patient notes as well as laboratory data, getting insight of patient feedback comments and triaging patients. Thus AI can find a place in not only clinical but also operational aspects of GI care.

Till now, AI has served as a valuable complementary tool for the clinicians and will nonetheless continue to do so but whether AI can replace the human touch completely is a serious question that puts all of us in a dilemma. It’s not a question that we can answer right now since there’s a lot of guidance that we need regarding the ethical and the regulatory aspects of AI in medicine.

## Conclusions

Current data suggests that AI models can reach a significant level of accuracy in rapidly diagnosing gastrointestinal pathologies by processing imaging and histopathologic data and in some cases, performs equal to or better than humans. Therefore, it is the need of the hour to perform randomized control trials to demonstrate the powers of a well-trained AI model which can then be adopted widespread to assist in accurate and rapid diagnosis and screening of various pathologies. Most of the studies have been done on the use of AI in processing image data which can be in the form of radiological scans like CT scans and MRIs, pictures or videos of endoscopies and histopathology. As such, AI can prove to be a very efficient and reliable assistant, aiding clinicians in the diagnosis and prioritizing which studies to look at first. However, we are far from AI models completely replacing humans. The applications of AI are not limited to diagnosis based on imaging data, but can also be applied to other aspects of patient care like triaging patients and risk assessment. We can conclude that AI is going to be an integral part of GI and healthcare in general in the near future.
